# Automated Assessment of Cardiovascular Sufficiency Using Non-Invasive Physiological Data

**DOI:** 10.3390/s22031024

**Published:** 2022-01-28

**Authors:** Xinyu Li, Michael R. Pinsky, Artur Dubrawski

**Affiliations:** 1 Auton Lab, School of Computer Science, Carnegie Mellon University, Pittsburgh, PA 15213, USA; awd@cs.cmu.edu; 2 Department of Critical Care Medicine, School of Medicine, University of Pittsburgh, Pittsburgh, PA 15213, USA; pinsky@pitt.edu

**Keywords:** fluid resuscitation, cardiovascular sufficiency, machine learning, physiological data, non-invasive monitoring

## Abstract

For fluid resuscitation of critically ill individuals to be effective, it must be well calibrated in terms of timing and dosages of treatments. In current practice, the cardiovascular sufficiency of patients during fluid resuscitation is determined using primarily invasively measured vital signs, including Arterial Pressure and Mixed Venous Oxygen Saturation (SvO2), which may not be available in outside-of-hospital settings, particularly in the field when treating subjects injured in traffic accidents or wounded in combat where only non-invasive monitoring is available to drive care. In this paper, we propose (1) a Machine Learning (ML) approach to estimate the sufficiency utilizing features extracted from non-invasive vital signs and (2) a novel framework to address the detrimental impact of inter-patient diversity on the ability of ML models to generalize well to unseen subjects. Through comprehensive evaluation on the physiological data collected in laboratory animal experiments, we demonstrate that the proposed approaches can achieve competitive performance on new patients using only non-invasive measurements. These characteristics enable effective monitoring of fluid resuscitation in real-world acute settings with limited monitoring resources and can help facilitate broader adoption of ML in this important subfield of healthcare.

## 1. Introduction

Substantial loss of blood can lead to hemorrhagic shock, organ dysfunctions, and death, if not treated promptly and effectively [1,2,3]. However, if patients are over-resuscitated, the excessive administration of fluids or blood can put an extra burden on the patient’s organs and result in poor outcomes of gravity equal to or even worse than that of under-resuscitation [4,5,6]. Hence, accurate judgement on the circulatory sufficiency of the subject during resuscitation would allow better titration of fluids and vasoactive drugs, as well as help determine when further treatment is unwarranted to sustain tissue vitality [7,8,9].

Within the context of resuscitation from critical illness associated with cardiovascular insufficiency, we introduce the term “Cardiovascular Sufficiency” to refer to the state of cardiovascular parameters consistent with adequate blood flow to meet the metabolic demands of the body without being in overt failure. Such a state may exist because the subject is on mechanical ventilatory support, receiving vasoactive drug infusion or boluses of fluids. However, once in a sufficient state, no additional changes in care are needed. Clinicians can readily define when a patient is in a cardiovascular insufficient state, and sufficiency occurs when not in that state. As an operational definition, cardiovascular sufficiency defines when additional resuscitative efforts or increases in resuscitative drug support are not needed.

Cardiovascular sufficiency can be achieved by the return of normal end-organ function. However, such clinically-relevant end-points usually take long time to manifest. Therefore, clinicians often continue aggressive resuscitative efforts beyond the levels needed to attain sufficiency, leading to complications associated with volume overload and vasoactive drug over-infusion. In the highly instrumented patients receiving cardiovascular resuscitation from circulatory shock, cardiovascular sufficiency can be temporarily defined as a state wherein Mean Arterial Pressure (MAP) is above some target level (usually >65 mmHg), reduced excessive sympathetic tone characterized by the absence of tachycardia (heart rate (HR) < 110/min), resolution of any of metabolic acidosis (often quantified by serum lactate levels < 2 mmol/dL), and adequacy of oxygen delivery to the tissues to meet their metabolic demand (usually approximated by a mixed venous oxygen saturation (SvO2) > 70%) [10,11]. Although MAP, HR, and serum lactate levels can be rapidly and continually assessed at the bedside in most hospitalized patients, estimates of SvO2 require highly invasive monitoring with a pulmonary artery catheter to sample pulmonary arterial blood. If estimates of SvO2 or overall cardiovascular sufficiency could be made continuously using non-invasive measures of physiologic time series data, then the current criteria for deciding the resuscitation sufficiency could be directly applied.

In this paper, we propose (1) a Machine Learning (ML) approach to predict the cardiovascular sufficiency at different time steps during the entire resuscitation horizon using only features extracted from non-invasive physiological data and (2) an analytic protocol that overcomes vulnerability of the resulting models to inter-patient variability.

For an ML approach to be successfully deployed in real-world healthcare applications, a common challenge to overcome is the inter-patient diversity [12]. The indigenous physiological variability among the patients in the training data can lead to harmful effects on the generalizability of the trained model on previously unseen patients. Reference to a personal baseline collected during the patients’ stable state can greatly help reduce the detrimental effects of heterogeneity among the patients and improve the performance of the downstream machine learning models [13,14,15]. However, for patients presenting in already acute states, e.g., trauma patients rushed in for care, there is no such luxury of observing the patients’ personal baseline information when they were stable. In this paper, we propose a novel framework, *Optimized Aggregation of Predictions*, to alleviate the need for personal baseline reference data by smartly aggregating the predictions made by the models with access to the personal baselines of previously seen patients. When applied to assess the resuscitation sufficiency of a new patient, the trained model will be deployed in combination with the personal baselines of previously seen patients to produce the final outcomes.

We experiment and evaluate our approaches using a porcine model of induced hemorrhagic shock and fluid resuscitation. Our results show that when using our proposed ML approach, we can achieve: (1) accurate prediction of resuscitation sufficiency using features derived from only non-invasive vital signs and (2) competitive performance on new patients without the requirement of accessing their own stable personal baselines. These two key advantages will allow our ML approach, once validated in humans, to be readily deployable in real-world acute out-of-hospital settings with limited monitoring resources.

## 2. Materials and Methods

### 2.1. Data

A laboratory porcine model of induced hemorrhagic shock and fluid resuscitation was utilized for our experiments. Sixteen healthy female pigs were sedated for induction, and all subjects had a pulmonary artery catheter (PAC) (Vigilance catheter; Edwards LifeSciences, Irvine, CA, USA) inserted via the internal jugular vein, a triple lumen 18-gauge catheter inserted into a femoral artery, and a large-bore introducer (8F) inserted into the femoral vein. The arterial pressure signal was simultaneously recorded on a LiDCOplus monitor (LiDCO, London, UK). Triplicate bolus thermodilution cardiac output was used to calibrate the LiDCO monitors and the PAC continuous cardiac output.

After the configuration, the animals were stabilized for 30 min to establish personal baselines, and then bled using a roller pump (Masterflex L/S easy-load II pump; Cole-Parmer, Vernon Hills, IL, USA) at a constant rate of 10 mL/min until their MAP decreased below 40 mmHg. Then, after a waiting period of 30 min, the fluid resuscitation started and then ended by the clinicians when the circulatory status was deemed sufficient, and evidence of cardiovascular stability had been observed. Physiological data at different granularities were collected in the process, including the beat-to-beat, non-invasive estimates of MAP, stroke volume variation (SVV), pulse pressure variation (PPV) using plethysmographic waveform analysis, and the high-resolution waveform data recorded at 250 Hz, including invasive arterial pressure, electrocardiogram (ECG), photo-plethysmography pulse oximetry, SvO2, SpO2, and airway pressure.

### 2.2. Labeling of Sufficiency

**Figure 1:**
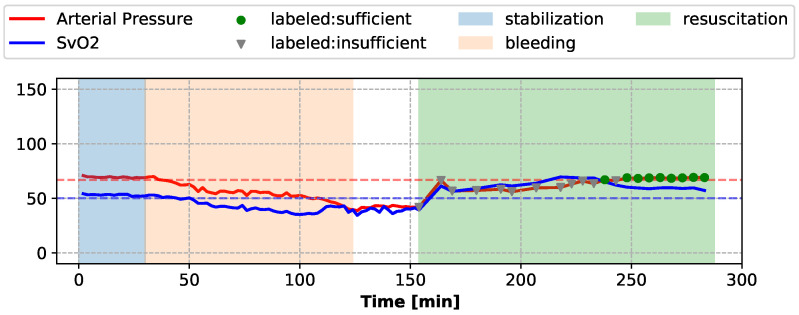
When the mean of Arterial Pressure and the mean of SvO2 (both invasively measured) are above the target values (dashed lines), the subject is labeled as “sufficient” at the given assessment time, or as “insufficient” otherwise.

During the resuscitation stage, sufficiency assessments were conducted every 5 min or 11 min, when a fluid bolus was administered (application of each fluid bolus took 6 min). Expert clinicians used the means of the invasively collected Arterial Pressure waveform and SvO2 waveform during the 2-min segments preceding each assessment to label the current sufficiency state of the subject. If the mean Arterial Pressure and the mean SvO2 were both above their respective target values, the subject was considered “sufficient” during this assessment segment, and “insufficient” otherwise. The target values of Arterial Pressure and SvO2 were determined by the mean and the standard deviations of the Arterial Pressure and SvO2 waveforms during the stable period of each individual subject. The sufficiency state labeling of one subject is shown in Figure 1 as an example.

In addition to the data collected during the 2-min assessment segments during the resuscitation period, we also sampled 2-min segments evenly from the stable, bleeding, and waiting stages for model training, and we labeled these segments using the same criteria. For each 2-min segment, moving windows of 20 s were extracted every 10 s, resulting in 11 time windows extracted from each segment. All such windows falling within the same segment shared the same sufficiency label with the segment. The descriptive statistics of the resulting dataset are provided in Appendix A.

### 2.3. Featurization

For each 20-s window, heart rate variability (HRV) measurements [16,17,18] and aggregated statistics were derived from the non-invasive ECG waveform, and beat-to-beat geometric features [19,20,21] were derived from the non-invasive photo-plethysmography pulse oximetry waveform. Beat-to-beat measures, including MAP, SVV, and PPV, which were directly extracted from the non-invasive beat-to-beat estimates, and HR and pulse transit time (PTT), which were derived from the non-invasive ECG and photo-plethysmography pulse oximetry waveforms, were also included in our feature set. For all the beat-to-beat features, the median, inter-quartile range (IQR), and linear slope were computed over all the heartbeats within the same window and used as aggregated features. We ended up with 42 features in total. The detailed description of the features is provided in Appendix B.

### 2.4. Normalization Using Personal Baseline

To mitigate the inter-subject variation during model training, we normalize each training subject’s data using its own personal baseline established from the subject’s stablization period data. For each training subject, each feature is normalized using its median and 90% range computed from the data points collected during the stable stage. Median and 90% range are used instead of mean and standard deviation, as commonly used in feature scaling, in order to prevent the potential negative effects of non-standard noise and outliers. The medians and the 90% ranges of all features are referred to as “normalization factors” in the following sections. If we have *d* features, the normalization factors of a subject will be a pair of vectors (m,r), $m\in R^{d},r\in R^{d}$. Given data $X\in R^{d}$ and (m,r), the normalized data $\bar{X}$ will be

(1) $$\bar{X}=\frac{\left(X-m\right)}{r}.$$

### 2.5. Optimized Aggregation of Predictions

**Figure 2:**
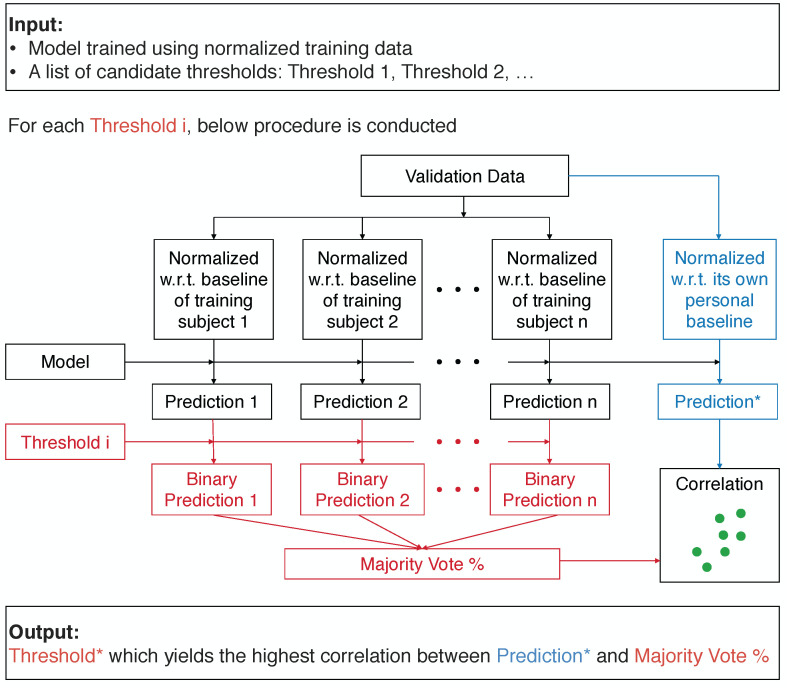
The Optimized Aggregation of Predictions framework. From a list of candidate thresholds, the one which maximizes the correlation between the predictions made by the trained model on the validation data normalized with reference to its own personal baseline (Prediction*, denoted by blue) and the aggregated binary predictions made by the same model on the validation data standardized using the normalization factors of different training subjects (Majority Vote %, denoted by red) is chosen to be used for converting the prediction scores to binary predictions on the test data.

To achieve accurate predictions on new patients with no available personal baseline information, we propose a novel framework, *Optimized Aggregation of Predictions*, which aggregates the binary predictions made by the model trained using the normalized data of training subjects. The threshold used for converting the prediction scores to binary predictions is tuned on the validation set. From a list of thresholds, the threshold which maximizes the correlation between the predictions made by the trained model on the validation data normalized with reference to its own personal baseline, and the aggregated binary predictions made by the same model on the validation data standardized using the normalization factors of different training subjects is selected. By using the normalization factors of each training subject, different “normalized” versions of the validation data are generated, and different binary predictions will be made by the same model on these different “normalized” versions. Then, these binary predictions are aggregated by taking the percentage of the positive predictions, in a majority voting scheme. The block diagram of this procedure is shown in Figure 2. The model can be any type of machine learning algorithm with a numeric score output, and we used the Random Forest model [22] in our experiments. The list of candidate thresholds was created by uniformly sampling from the unique prediction scores made on the validation data points by the model.

**Figure 3:**
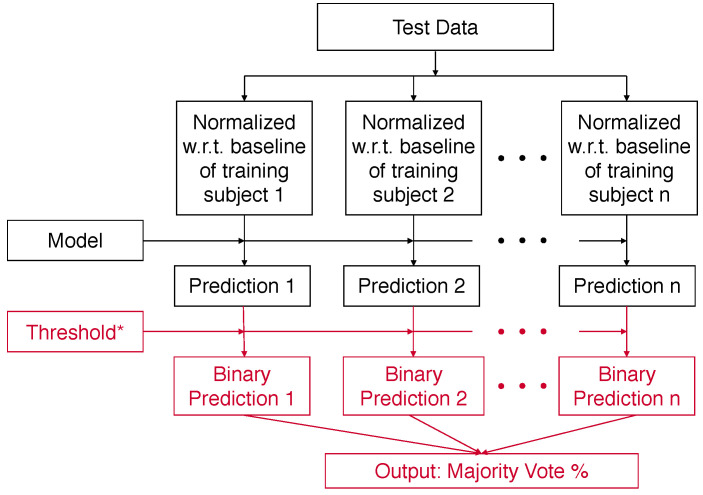
The predictions for the test data using the threshold chosen via optimization performed using the validation data as shown in Figure 2.

At test time, the different “normalized” versions of the test data are generated in a similar fashion using the normalization factors of the training subjects. The single optimal threshold selected on the validation set will be used to convert the prediction scores for each “normalized” version of the test data to binary predictions, and the majority vote % will be the final prediction output, as shown in Figure 3. In the medical context, this voting output has a clinically relevant interpretation as it directly informs the clinicians of how confident the model is by measuring how many previously seen patients have voted for the positive prediction made for the current subject.

### 2.6. Model Training and Evaluation

We trained a Random Forest model using Python *sklearn* library [23] over extracted moving windows to classify the sufficiency state, with sufficient windows belonging to the positive class and insufficient the negative class. To accommodate our relatively small cohort, we trained and evaluated our model using a leave-one-subject-out cross-validation protocol, i.e., during each training iteration, the moving windows of one single subject were held-out as the test set, while the data of all the other subjects were used for training and validation. When tuning the binary prediction threshold using the *Optimized Aggregation of Predictions* framework, we conducted an inner-loop leave-one-subject-out cross-validation to select the optimal threshold, i.e., the threshold which yielded the highest average correlation across all validation subjects was chosen and utilized on the test subject. To make our evaluation as similar to the real-world clinical scenarios as possible, we trained our model using all data points during all stages but only evaluated the performance on the resuscitation stage. The data during the resuscitation periods of 4 subjects were corrupted due to device issues in the laboratory experiments. After the verification with the clinicians, their resuscitation data were removed, and only 12 out of 16 subjects were used in validation and testing.

## 3. Results

### 3.1. Moving Window Classification Performance

**Figure 4:**
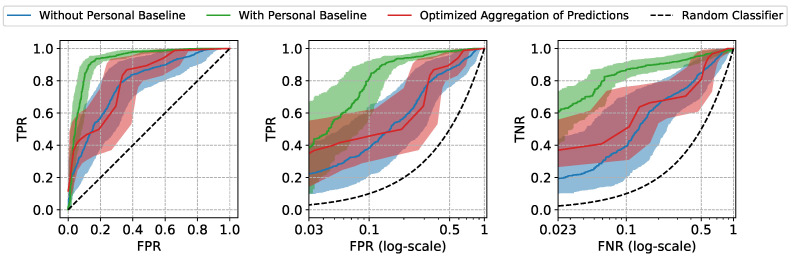
The mean and the standard error bands of the ROC curves of three different approaches for resuscitation sufficiency prediction. The False Positive Rate (FPR) and the False Negative Rate (FNR) are scaled logarithmically in the middle and right plots to emphasize the performance at the clinically relevant low prediction errors settings.

**Figure 5:**
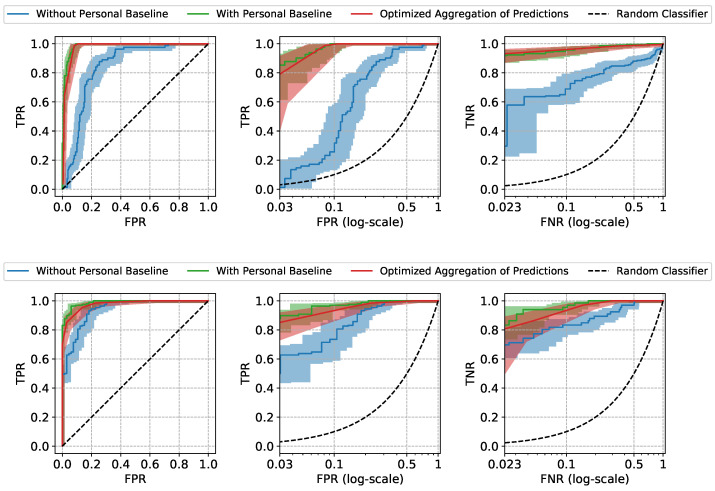
ROC curves of two example test subjects. The 95% confidence intervals are computed using the Wilson interval scores.

The Receiver Operating Characteristic (ROC) curves and the performance metrics of our approach are shown in Figure 4 and Table 1. The mean and the standard error (Due to the high inter-subject variation in our data, standard error bounds are provided for the aggregated results instead of 95% confidence intervals) of the ROC curves and the metrics are computed by aggregating the results of leave-one-subject-out cross-validation. The model trained and tested using only non-normalized features (referred to as “Without Personal Baseline”) and the model trained and tested using only normalized features (referred to as “With Personal Baseline”) are provided as references, as the former intuitively represents the worst performance expected when the personal baselines from neither the training subjects nor the test subjects are available, and the latter intuitively represents the best performance expected when there is the luxury to observe the stable personal baselines of the new patients. As expected, when the personal baselines of the test subjects are available, the “With Personal Baseline” model achieves the highest Area Under ROC curve (AUROC) scores. When there is no access to the personal baselines of the test subjects, our *Optimized Aggregation of Predictions* approach is able to achieve a comparable AUROC to the “With Personal Baseline” model and a better performance than the “Without Personal Baseline” model. In addition to the aggregated results, we also provide the ROC curves of two individual test subjects in Figure 5 as examples, with the 95% confidence intervals computed using the Wilson score interval [24].

The two types of prediction errors, namely the false positives and the false negatives, can both lead to unfavorable patient outcomes. To emphasize the model’s performance at the clinically relevant low error settings, the True Positive Rate (TPR) at low False Positive Rate (FPR) and the True Negative Rate (TNR) at low False Negative Rate (FNR) are also provided. The lowest FPR and FNR are determined by the minimum frequency of insufficient (negative) and sufficient (positive) windows in our test subjects (the details are provided in Appendix A). At the low FPR region, our approach achieves TPR of 0.343, very close to the highest TPR 0.387 achieved by “With Personal Baseline” model, which means that our approach is able to correctly identify 34.3% of all the sufficient moving windows (the positive class) in test subjects, while only misclassifying 3 insufficient moving windows as sufficient out of 100 such predictions, on average. The false predictions of sufficiency (false positive errors) may wrongly advise the stopping of the resuscitation and, thus, lead to under-resuscitation. If, in practice, a decision threshold is chosen accordingly, at low FPR to avoid such unfavorable outcomes, our approach is still able to identify 34.3% sufficient windows correctly. Similarly, if a decision threshold is chosen at low FNR region to avoid the misclassifications for sufficient windows (false negative errors) which may lead to over-resuscitation, our approach is able to correctly classify 35.8% insufficient windows, much higher than the 19.5% achieved by the “Without Personal Baseline” model, while only giving 2.3 false alerts of insufficiency out of 100 such predictions, on average. By comparing the performance at the operationally useful low FPR and FNR regions, we demonstrate the high practical utility potential of the proposed approach when the stable personal baselines of new patients are not available, as is often the case in urgent field care scenarios.

### 3.2. Cost-Optimal Decision Threshold Selection

When ML approaches are transitioned to real-world healthcare applications, clinicians would typically settle on using a single decision threshold, which, in our case, was used to determine the sufficiency state at any given time point during the resuscitation. We utilize the framework explained in Reference [25] to select the optimal decision threshold by minimizing the expected cumulative costs of classification errors.

If we can estimate the relative frequencies of the positive and the negative examples in the test data (denoted by *p* and *n*, respectively), as well as the expected ratio of the average unit cost of each false positive error ($C_{FP}$) to the average unit cost of each false negative error ($C_{FN}$), we can draw an *iso-performance* line in the ROC plane, with the slope computed as $\frac{n}{p}\cdot \frac{C_{FP}}{C_{FN}}$. The points on the same *iso-performance* line will represent a collection of hypothetical decision thresholds with the same expected misclassification costs. At the same time, a feasible decision threshold will also reside on the ROC curve of the trained model. Thus, the cost-optimal decision threshold corresponds to the point on the ROC curve which is tangential to the *iso-performance* line whose slope is determined by the estimated *p*, *n*, $C_{FP}$, and $C_{FN}$.

The ratio $\frac{C_{FP}}{C_{FN}}$ can be determined by the domain knowledge for the specific clinical task. For our task, false positive errors correspond to misclassifying insufficient as sufficient which may lead to under-resuscitation, while false negative errors, corresponding to misclassifying sufficient as insufficient, may lead to over-resuscitation. Since both are unfavorable outcomes, we provide the evaluation of three different settings: $\frac{C_{FP}}{C_{FN}}=1$ when both are equally costly, $\frac{C_{FP}}{C_{FN}}=3$ when under-resuscitation is threefold more unfavorable, and $\frac{C_{FP}}{C_{FN}}=\frac{1}{3}$ when over-resuscitation would be perceived three times more costly than the opposite type of error.

**Figure 6:**
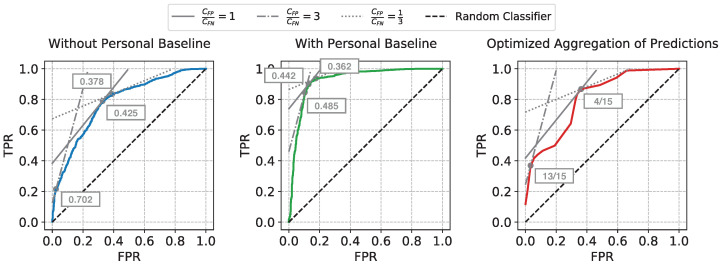
The *iso-performance* lines and the selected cost-optimal decision thresholds (shown in boxes) corresponding to the three different settings of $\frac{C_{FP}}{C_{FN}}$.

The same ROC curves from Figure 4 are used for this cost analysis. The *iso-performance* lines and the selected cost-optimal decision thresholds corresponding to the three settings of $\frac{C_{FP}}{C_{FN}}$ ratios are shown in Figure 6. The $\frac{n}{p}$ ratio is estimated by taking the average relative frequency between the insufficient windows and the sufficient windows across all test subjects. The decision thresholds for our *Optimized Aggregation of Predictions* approach are directly shown as the fraction of training subjects voting for sufficiency. As we have 16 subjects in total, and leave-one-subject-out cross-validation is used for evaluation, for each held-out test subject, 15 subjects are used for training. When the average unit cost of false positive errors is considered to be higher than the average unit cost of false negatives, i.e., under-resuscitation is more costly than over-resuscitation, a higher decision threshold will be chosen to lean towards prevent the more costly errors. On the contrary, a lower decision threshold will be selected to avoid missing of sufficiency when over-resuscitation is considered more costly ($\frac{C_{FP}}{C_{FN}}=\frac{1}{3}$). After the optimal decision threshold is chosen under each $\frac{C_{FP}}{C_{FN}}$ assumption, the numeric outputs of the approaches are converted to binary predictions, and the McNemar’s tests [26,27] are conducted to compare our approach against the two reference approaches. The moving windows from all test subjects are concatenated together for the McNemar’s tests.

**Figure 7:**
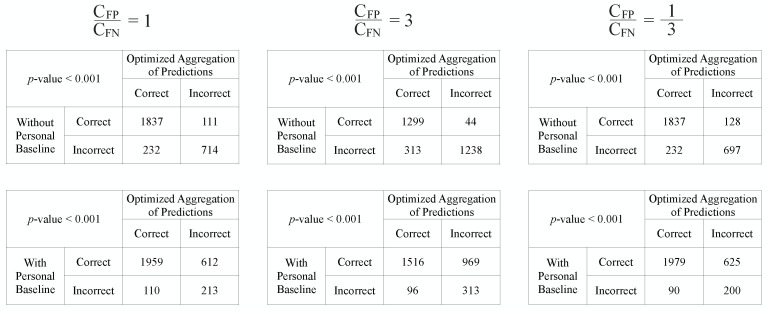
The contingency tables and the *p*-values for the McNemar’s tests.

The contingency tables and the *p*-values from McNemar’s tests are shown in Figure 7. From the 3 tables in the upper row, we can see that the proposed approach performs significantly better than the “Without Personal Baseline” approach with *p*-values < 0.001 across all 3 settings of $\frac{C_{FP}}{C_{FN}}$, as the number of mistakes our approach makes in the examples correctly predicted by the “Without Personal Baseline” approach (the upper right entries of McNemar’s contingency tables shown) is consistently lower than the number of mistakes that the “Without Personal Baseline” approach would make in the examples that our approach is able to correctly classify (the lower left entries in these tables). Based on the statistics in the 3 tables in the lower row, the “With Personal Baseline” approach still has the best performance. However, in real-world urgent acute settings when the stable personal baseline information is typically not available, the proposed approach is able to significantly improve the performance of the resuscitation sufficiency assessment model by leveraging the personal baselines of previously seen patients.

The $\frac{C_{FP}}{C_{FN}}$ ratio may vary across different healthcare sites or organizations due to the different tiers of care, varying implementation of the best practices of care, or even temporally varying availability of healthcare providers or other resources. With the proposed framework, which selects the optimal decision threshold by minimizing the expected costs of misclassifications, the same trained model can be tuned to optimize the operational performance under varying assumptions of relative costs of errors specific to the circumstances where the model is going to be used, without the requirement of re-training it. This evaluation framework provides a practical solution to the scenario when the main difference between the various application scenarios is due to the varying costs of prediction errors, rather than the differences between the underlying distributions of the clinical data with which the model is trained.

## 4. Discussion

In this paper, we proposed a machine learning approach to model the cardiovascular sufficiency state of patients undergoing resuscitation with several specific designs to bridge the gaps between our method and its potential implementation in real-world field and urgent care practice. Our main contributions include:

- Using only non-invasively collected, easily-available physiological data, the level of cardiovascular sufficiency during resuscitation can be accurately determined, which enables the proposed approach to be implemented in a wide range of real-world applications with limited or no access to invasive monitoring.
- The proposed general framework, *Optimized Aggregation of Predictions*, can alleviate the need for stable personal baseline information of the new patients, the main source of non-informative variability in hemodynamic monitoring data. It accomplishes that by leveraging personal baselines of previously seen patients, even when facing high inter-patient diversity in the data. This allows the proposed framework to be readily implementable in real-world field and urgent care settings, including care for patients presenting in acute states, for whom we do not have access to the vital sign data measured when they were stable.
- We demonstrated how to choose the optimal set-point of the resuscitation sufficiency assessment models by combining ROC curve analysis and the expected relative cost ratios of prediction errors, to achieve the optimal performance in various application scenarios that differ in relative error cost ratios due to operational factors, such as different tiers or implementations of care, or availability of personnel and other resources.

The main limitation of our work lies in the highly controlled nature of the experiment design of the laboratory porcine model, and the relatively small number of subjects in our cohort, though precautionary techniques, such as leave-one-subject-out cross-validation, have been utilized to mitigate the risk of over-fitting the models.

Our future directions include adapting the presented work to larger and more complicated human patient databases collected in intensive care units in hospitals and similar data collected in emergency medicine scenarios in the field and during medical evacuation of wounded or injured subjects. We are also adopting our approach to serve as a fully automated closed-loop resuscitation control system capable of switching between aggressive and maintenance resuscitation modes, or turning it off completely when appropriate, based on evidence observed in non-invasively collected vital signs data in real-time.

## Figures and Tables

**Table 1:** The mean and the standard error intervals of the AUC scores, True Positive Rate (TPR) at low False Positive Rate (FPR), and True Negative Rate (TNR) at low False Negative Rate (FNR) of three different approaches for resuscitation sufficiency prediction.

Approach	AUROC	TPR at FPR = 0.030	TNR at FNR = 0.023
Without Personal Baseline	0.892(0.873,0.911)	0.223(0.098,0.412)	0.195(0.102,0.439)
With Personal Baseline	0.947(0.930,0.963)	0.387(0.101,0.670)	0.607(0.399,0.737)
Optimized Aggregation of Predictions	0.929(0.908,0.950)	0.343(0.134,0.551)	0.358(0.261,0.542)

## Data Availability

The original raw data are privately owned by the University of Pittsburgh, while its derivatives have been developed by and are in possession of Carnegie Mellon University, and neither can be shared publicly due to legal restrictions.

## Appendix A. Descriptive Statistics of the Dataset

Each subject in our cohort experienced several stages during the laboratory experiments: stabilization, bleeding, waiting, and resuscitation, as stated in Section 2.1. Our model was trained using all the moving windows extracted from both the pre-resuscitation stages (stabilization, bleeding, and waiting) and the resuscitation stage, while the performance evaluation was conducted using only the resuscitation stage data. The cumulative descriptive statistics of data observed at the different stages of experiment are provided in Table A1.

We take the maximum of the lowest FPR and FNR achievable by each test subject as the minimum FPR and FNR in the ROC curves in Figure 4 and Figure 5. Based on the actual frequency of the insufficient (negative) and sufficient (positive) windows in each subject, the maximum lowest FPR is $1/\min\;(\mathrm{number}\;\mathrm{of}\;\mathrm{insufficient}\;\mathrm{windows})=\frac{1}{33}=0.030$, and the maximum lowest FNR is $1/\min\;(\mathrm{number}\;\mathrm{of}\;\mathrm{sufficient}\;\mathrm{windows})=\frac{1}{43}=0.023$.

**Table A1:** The statistics of the number of moving windows in different stages.

Class	Statistics	Number of Moving Windows in Each Subject	
		Pre-Resuscitation	Resuscitation
Sufficient (Positive class)	mean ± standard error	257.33±27.13	139.50±18.05
	min	195	43
	max	550	241
Insufficient (Negative class)	mean ± standard error	693.75±42.49	101.67±16.99
	min	495	33
	max	900	220

## Appendix B. Featurization

The features we derived from the non-invasive vital signs are described in Table A2. The HRV features were computed according to Reference [28]. For the HRV and aggregated statistics features, there is only 1 measurement for each 20-s moving window. For the beat-to-beat features, we computed the median, IQR, and linear slope over all the heartbeats falling within the same window, resulting in 3 aggregated measurements for each beat-to-beat feature. With 9 HRV and aggregated statistics features and 11 beat-to-beat features, we ended up with 9+11×3=42 features in total.

**Table A2:** The features derived from non-invasive vital signs.

Source Vital Sign	Feature Name	Feature Type
Photo-plethysmography Waveform	Systolic Amplitudes	Beat-to-Beat
Photo-plethysmography Waveform	Peak-to-Peak Interval	Beat-to-Beat
Photo-plethysmography Waveform	Pulse Interval	Beat-to-Beat
Photo-plethysmography Waveform	Upstroke Time	Beat-to-Beat
Photo-plethysmography Waveform	Beat Skewness [29,30]	Beat-to-Beat
ECG Waveform	HR	Beat-to-Beat
ECG Waveform	Standard Deviation of the NN Interval (SDNN)	HRV
ECG Waveform	Square Root of the Mean Squared Differences of Successive NN Intervals (RMSSD)	HRV
ECG Waveform	Very Low Frequency (VLF)	HRV
ECG Waveform	Low Frequency (LF)	HRV
ECG Waveform	High Frequency (HF)	HRV
ECG Waveform	Normalized Low Frequency	HRV
ECG Waveform	Normalized High Frequency	HRV
ECG Waveform	LF/HF	HRV
ECG Waveform	Approximate Entropy	Aggregated Statistics
Photo-plethysmography Waveform, ECG Waveform	Pulse Transit Time (PTT)	Beat-to-Beat
Non-invasive Beat-to-Beat Estimates	Mean Arterial Pressure (MAP)	Beat-to-Beat
Non-invasive Beat-to-Beat Estimates	Stroke Volume Variation (SVV)	Beat-to-Beat
Non-invasive Beat-to-Beat Estimates	Pulse Pressure Variation (PPV)	Beat-to-Beat
Non-invasive Beat-to-Beat Estimates	Dynamic Arterial Elastance (Eadyn, = PPV/SVV) [31]	Beat-to-Beat
